# Short-chain fatty acid concentrations in the incidence and risk-stratification of colorectal cancer: a systematic review and meta-analysis

**DOI:** 10.1186/s12916-022-02529-4

**Published:** 2022-10-03

**Authors:** Ehsan Alvandi, Wilson K. M. Wong, Mugdha V. Joglekar, Kevin J. Spring, Anandwardhan A. Hardikar

**Affiliations:** 1 Diabetes and Islet Biology Group, School of Medicine, Western Sydney University, Campbelltown, NSW Australia; 2 Medical Oncology Group, Ingham Institute for Applied Medical Research, Liverpool, NSW Australia; 3 Translational Health Research Institute, Western Sydney University, Campbelltown, NSW Australia; 4 South-West Sydney Clinical Campuses, UNSW Medicine & Health, Sydney, NSW Australia; 5 Liverpool Clinical School, School of Medicine, Western Sydney University, Liverpool, NSW Australia; 6 Department of Science and Environment, Roskilde University Copenhagen, Roskilde, Denmark

**Keywords:** Colorectal cancer, Adenoma, Short-chain fatty acid, Incidence, Risk, Meta-analysis

## Abstract

**Background:**

The beneficial role of gut microbiota and bacterial metabolites, including short-chain fatty acids (SCFAs), is well recognized, although the available literature around their role in colorectal cancer (CRC) has been inconsistent.

**Methods:**

We performed a systematic review and meta-analysis to examine the associations of fecal SCFA concentrations to the incidence and risk of CRC. Data extraction through Medline, Embase, and Web of Science was carried out from database conception to June 29, 2022. Predefined inclusion/exclusion criteria led to the selection of 17 case-control and six cross-sectional studies for quality assessment and analyses. Studies were categorized for CRC risk or incidence, and RevMan 5.4 was used to perform the meta-analyses. Standardized mean differences (SMD) with 95% confidence intervals (CI) were calculated using a random-effects model. Studies lacking quantitation were included in qualitative analyses.

**Results:**

Combined analysis of acetic, propionic, and butyric acid revealed significantly lower concentrations of these SCFAs in individuals with a high-risk of CRC (SMD = 2.02, 95% CI 0.31 to 3.74, *P* = 0.02). Additionally, CRC incidence was higher in individuals with lower levels of SCFAs (SMD = 0.45, 95% CI 0.19 to 0.72, *P* = 0.0009), compared to healthy individuals. Qualitative analyses identified 70.4% of studies reporting significantly lower concentrations of fecal acetic, propionic, butyric acid, or total SCFAs in those at higher risk of CRC, while 66.7% reported significantly lower concentrations of fecal acetic and butyric acid in CRC patients compared to healthy controls.

**Conclusions:**

Overall, lower fecal concentrations of the three major SCFAs are associated with higher risk of CRC and incidence of CRC.

**Supplementary Information:**

The online version contains supplementary material available at 10.1186/s12916-022-02529-4.

## Background

According to the Global Cancer Incidence, Mortality and Prevalence (GLOBOCAN) 2020 report, colorectal cancer (CRC) is the third most commonly diagnosed cancer (10% of all diagnosed cancers) and the second (9.4%) leading cause of cancer-related death [1]. It has been estimated that the overall risk of CRC in all age groups will increase 60% worldwide by 2030, leading to more than 1.1 million deaths and 2.2 million new cases [2]. Colorectal cancer develops from precursor lesions collectively known as colorectal adenomas (CRA), in the form of adenomatous polyps or to a lesser extent (10–20%) serrated polyps [3, 4]. It is a heterogeneous disease and environmental factors have a potential impact on the development of CRC, among which diet is a risk factor [4–6]. According to several meta-analyses, high consumption of processed and unprocessed meat is related to high CRC risk [7, 8], and high fiber intake is suggested as a protective factor against CRC progression and incidence [9–11].

The effect of diet on colonic health is partly mediated through alteration of gut microbiota composition, diversity, and metabolism [6, 12]. Gut microbiota constitutes the largest community of commensal microorganisms in the body, which mainly resides in the lower small intestine and colon [6, 12, 13]. The gut microbiota-derived metabolites are in constant crosstalk with colonocytes, and short-chain fatty acids (SCFAs) make up a large group of these metabolites [6, 12, 13].

Short-chain fatty acids are small molecules generated via the fermentation of dietary fibers by gut microbiota. Acetic, propionic, and butyric acid constitutes the majority of colonic SCFA content [14, 15] and the beneficial anti-inflammatory and anti-carcinogenic effects of dietary fibers on colonocytes are mediated through these SCFA molecules [16, 17]. Among the three major SCFA molecules, butyric acid is also considered as one of the main energy sources for colonocytes [12, 14, 17]. Therefore, alteration in SCFA levels could impact the colonic health and predisposition of colonocytes to aberrant proliferation and tumor formation [15, 16].

Several studies have assessed fecal SCFA concentration in patients with colorectal carcinoma or adenoma [18–34]. However, due to variable results, the conclusive evaluation of SCFA profiles from CRC patients versus healthy subjects is lacking. In addition, other studies have compared SCFA concentration within healthy individuals from various countries and ethnic groups with the highest and lowest prevalence of CRC; although with inconsistent results [35–40].

Therefore, systematic analyses designed to better understand the link between SCFA concentration in CRC risk and incidence is highly desired. We divided our analyses on the available evidence into two broad categories: (1) CRC-risk and (2) incidence. We aimed to systematically analyze the results of all primary observational human studies, which measured fecal SCFA levels in “at-risk” individuals or in CRC patients. In the CRC risk category, the focus was on at-risk individuals, which was further sub-divided into two groups based on (1a) studies that analyzed clinical data (presence of colorectal adenomas) or (1b) those that assigned CRC risk based on non-clinical evaluation of study participants (ethnic background or location). The CRC incidence category included studies that compared fecal SCFA levels in individuals with clinically diagnosed CRC and healthy individuals. Our results underline the potential association of the three major SCFA molecules (acetic, propionic, and butyric acid) with CRC risk and incidence.

## Methods

We used Preferred Reporting Items for Systematic Reviews and Meta-Analyses (PRISMA) 2020 guideline [41, 42] to systematically search and extract data from primary human studies with SCFA measurement in CRC risk or incidence.

### Database search

The Medline, Embase, and Web of Science database search was performed for articles involving human subjects that are in English from database conception until June 29, 2022. The details of the search keywords and strategies utilized in Ovid and Web of Science are available in the Additional file 1: Supplementary methods.

### Eligibility criteria

All the records, including abstracts, were imported to EndNote X9 (Clarivate Analytics, Toronto, Canada). Duplicate records were first removed. The records were then filtered using EndNote’s built-in search tool for the following criteria: (i) searching for concentration*, level*, quanti*, measure*, assess*, evaluat*, estimat*, calculat*, mmol, and μmol as the inclusion criteria to capture studies which reported the SCFA measurement based on these terms, and (ii) searching for mouse, mice, murine, rats, conference, ethyl acetate (EtOAc), and phorbol as the exclusion criteria to exclude rodent studies, conference proceedings, and studies that have stated the use of any unrelated chemicals (such as EtOAc and 12-O-Tetradecanoylphorbol-13-acetate) - the asterisk symbol (*) applied was to include all the variations of the search terms. The abstracts of the remaining records were then screened to exclude reviews, methodology, human studies not related to SCFAs in CRC or CRA, and non-human studies (i.e., in vitro or other non-rodent animal studies), to identify the human studies on SCFA measurement in CRC or CRA. The full text of the remaining (*n* = 57) records were then screened to include only the observational studies which have measured fecal SCFA concentration. A final set of 23 observational studies qualified for further data extraction, quality assessment, and statistical analyses.

### Data extraction and quality assessments

The data and additional details available for analysis (such as study subjects and SCFA levels) from the finalized primary studies were extracted and added to an Excel worksheet. The Newcastle-Ottawa Scale (NOS) [43] was used as a standard tool for quality assessment of 17 case-control studies in the selection, comparability, and exposure categories, to provide a score range between 0 and 9 (≤ 6, 7–8, and 9 indicate high, medium, and low risk of bias, respectively) [42]. Evaluation of six cross-sectional studies was performed using the Joanna Briggs Institute (JBI) Critical Appraisal Checklist tool [44], as recommended [45].

### Statistical analyses

Review Manager (RevMan) software version 5.4 (Cochrane, Copenhagen, Denmark) was used to analyze the quantitative fecal SCFA concentration data, which were available in 10 of the final 23 observational studies (9 of 17 case-control, plus 1 of 6 cross-sectional studies). The fecal concentration of acetic, propionic, or butyric acid was considered as the subgroups. Before data entry, SEM or 95% CI upper and lower bound values were converted to SD. Due to variation in the reported SCFA concentration units between different papers, standardized mean difference (SMD) was selected as a measure of effect size for each study. The statistical heterogeneity among studies was calculated using *χ*^2^ and *I*^2^ tests and a *P*-value of 0.05 was considered significant [46]. To normalize the use of different SCFA measurement methods, a random-effects model was applied to analyze the pooled effect size and *P*-value for each SCFA molecule in each subgroup. One overall effect size and *P*-value of combined acetic, propionic, and butyric acid were also calculated. In all analyses, the effect size was reported with 95% confidence intervals, and the *P*-value < 0.05 was considered significant. Furthermore, the fixed-effect model was also applied in the case of non-significant heterogeneity of *I*^2^ < 50 [46]. All the data conversions, as well as qualitative and quantitative analyses, were validated by the second team member and confirmed by the senior authors.

One study [34] reported values that could not be converted to mean and SD for the meta-analysis and was therefore only included in our qualitative analysis. Another study [35] reported the numeric values of butyric acid concentration and other SCFA molecules in graphs and hence was included in both quantitative (for butyric acid) and qualitative data (for acetic acid, propionic acid and total SCFA). Therefore, in addition to studies in which the fecal SCFA concentration was presented using graphs (with no reported actual values), 14 of 23 studies were considered as qualitative studies (8 of 17 case-control, plus all 6 cross-sectional studies—including Ocvirk et al. 2020). The outcome of analyses from these qualitative studies was plotted as stacked bar charts, using Microsoft Excel (ver. 2016; Microsoft Corporation, Redmond, WA, USA).

## Results

### Study selection and quality assessment

**Fig. 1:**
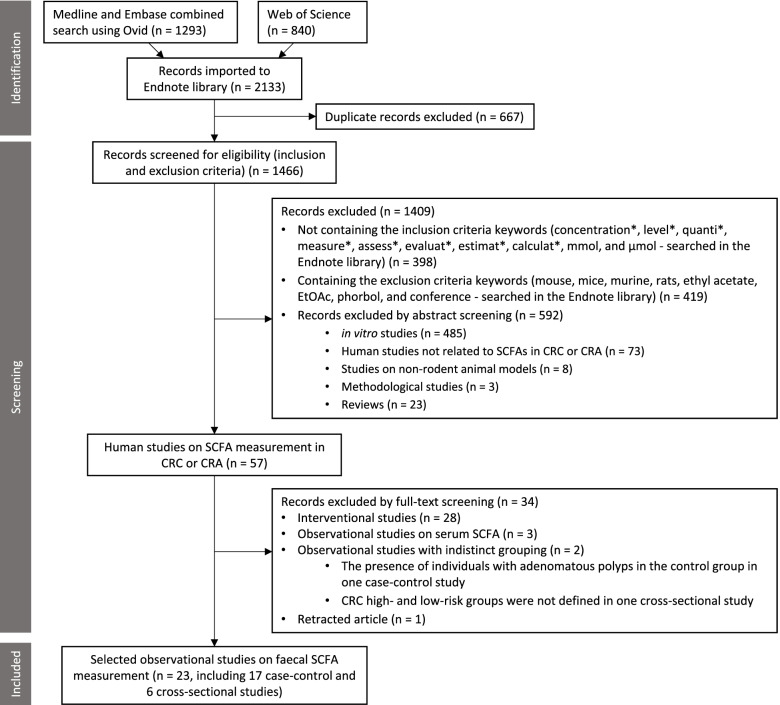
The PRISMA flowchart shows the selection process of the systematic review. The abstracts of all the studies were imported into Endnote from the indicated databases. SCFA, short-chain fatty acid; CRC, colorectal cancer; CRA, colorectal adenoma

The workflow on the identification and stepwise selection of the observational studies is presented in Fig. 1. Initially, a total of 2133 English language records obtained from searching through the three databases (Medline, Embase, and Web of Science) were imported to EndNote along with their abstracts. After removing duplicate records, the titles and abstracts of the remaining 1466 records were filtered and screened for eligibility as detailed in the Methods section. In total, 1409 records were excluded, of which most were in vitro studies. From the remaining 57 human studies, 34 studies were excluded. Of these 28 were interventional studies, three were observational studies on serum SCFA [47–49], two studies had indistinct grouping (one case-control study with the presence of individuals with adenomatous polyps in the healthy control group [50] and one cross-sectional study with no clear definition of CRC high- and low-risk group [51]), and one retracted observational study [52].

Finally, 17 case-control and 6 cross-sectional studies were selected for data extraction and analysis. Table 1 summarizes the characteristics of these observational studies. The results of quality assessment using NOS and JBI tools on case-control and cross-sectional studies are provided in Additional file 1: Tables S1 and S2, respectively.

**Table 1.**
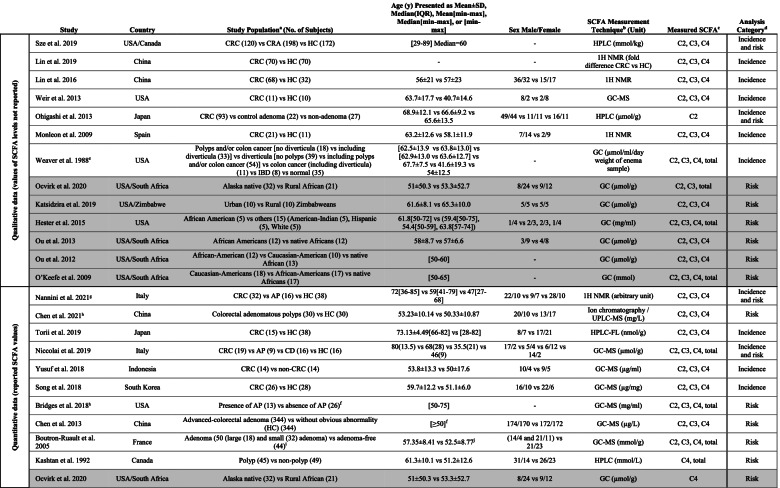
Characteristics of the selected studies. Cross-sectional studies are highlighted in gray, and case-control studies are not highlighted

^a^*CRC* colorectal cancer, *AP* adenomatous polyposis, *CD* celiac disease, *CRA* colorectal adenoma, *HC* healthy controls, *IBD* inflammatory bowel disease. ^b^*GC-MS* gas chromatography-mass spectrometry, *HPLC* high-performance liquid chromatography, *FL* fluorescence, *1H NMR 1H* nuclear magnetic resonance spectroscopy, *UPLC-MS* ultra-performance liquid chromatography-tandem mass spectrometry, *GLC* gas-liquid chromatography. ^c^*C2* acetic acid, *C3* propionic acid, *C4* butyric acid. ^d^Refer to the text for the definition of CRC risk and incidence category. ^e^Values in this paper were measured on enema samples, not feces. Therefore, they used in qualitative analysis. ^f^More details are provided in the article. ^g^Removed from quantitative analysis as the reported SCFA values could not be converted to mean and SD. ^h^Removed from meta-analysis due to insufficient data on SCFA measurement method. ^i^SCFAs were measured in only a subset of these subjects (*n* = 25 large/small adenoma and* n* = 23 adenoma-free). ^j^Combined values of males and females

### Stratifications based on CRC risk or incidence

Studies listed in Table 1 are presented based on the type of data provided (qualitative or quantitative) and CRC risk and/or incidence. Among the 17 case-control studies (not highlighted in Table 1), 8 studies comparing CRC cases and healthy control subjects were allocated to the CRC incidence category, 5 studies comparing individuals with CRA and healthy controls assigned to the CRC risk category, and the remaining 4 studies were included in both incidence and risk categories since they compared CRC patients, CRA individuals, and healthy subjects. All 6 cross-sectional studies (highlighted gray in Table 1) comparing populations with high- versus low-risk of CRC were allocated to the risk category. Therefore, the CRC incidence and risk category included 12 and 15 studies, respectively (Table 1). For each study, the details of the measured SCFA and CRC risk and/or incidence grouping are provided in Additional file 1: Table S3. Some studies reported total SCFA concentration in addition to the individual (acetate, propionate, and butyrate) SCFAs.

The primary studies analyzed in this systematic review were performed in various countries and ethnic groups. Age was matched in some of the studies [18, 24, 28, 38–40], although the male-to-female ratio was not similar between the study groups in most studies (Table 1). The SCFA concentrations were measured using different techniques, such as gas chromatography, liquid chromatography, gas-liquid chromatography and ^1^H nuclear magnetic resonance spectroscopy.

### Data analyses

**Fig. 2:**
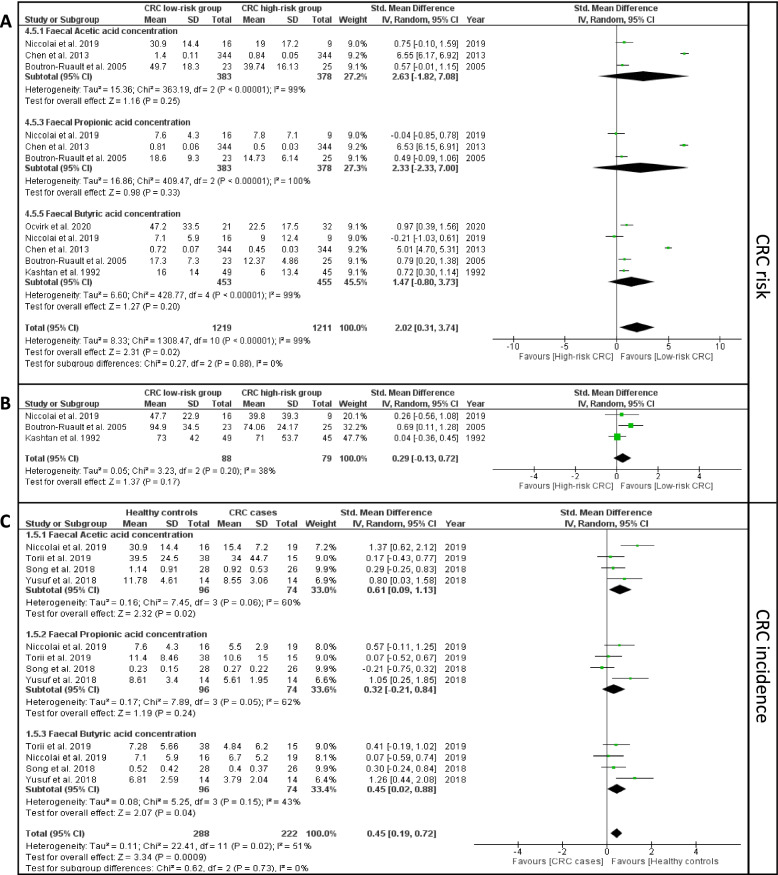
Forest plots representing the meta-analyses of the fecal concentrations of **A** acetic, propionic, and butyric acid in CRC risk category; **B** total SCFA in CRC risk category; and **C** acetic, propionic, and butyric acid in CRC incidence category. Note that in **B**, the total SCFA indicates the collection of all the SCFA molecules—not only acetic, propionic, and butyric acid

The meta-analysis of the quantitative data extracted from the 10 selected studies [18–22, 26, 28, 30, 33, 35] are presented in Fig. 2. In the risk category (Fig. 2A. B), two studies [19, 20] were excluded from the meta-analysis due to the lack of sufficient details of the methods used for SCFA measurement from stool samples. In CRC risk meta-analysis, the effect size of each of the three SCFAs was not statistically significant; however, their combined effect size was significantly higher in low risk compared to high-risk CRC (SMD = 2.02, 95% CI 0.31 to 3.74, *P* = 0.02, Fig. 2A). The effect size of total SCFA concentration was not statistically significant in the low- vs high-risk group (Fig. 2B).

In the CRC incidence analysis (Fig. 2C), the fecal concentrations of acetic acid (SMD = 0.61, 95% CI 0.09 to 1.13, *P* = 0.02) and butyric acid (SMD = 0.45, 95% CI 0.02 to 0.88, *P* = 0.04) were significantly higher in the healthy control compared to CRC cases. In addition, the combined effect size of acetic, propionic, and butyric acid remained significant between CRC cases and healthy controls (SMD = 0.45, 95% CI 0.19 to 0.72, *P* = 0.0009, Fig. 2C).

Furthermore, the *I*^2^ heterogeneity index was in the “moderate” range (30 to 60%) [46] for the meta-analysis of total SCFA concentration in CRC risk (Fig. 2B) and butyric acid in CRC incidence (Fig. 2C) category. Therefore, we performed another meta-analysis using the fixed-effect model on the same data instead of the random-effect model presented in Table 2. This resulted in a more pronounced difference in butyric acid concentration between CRC cases and healthy controls (SMD = 0.42, 95% CI 0.1 to 0.74, *P* = 0.009). The results of the fixed-effect model meta-analyses are presented in Additional file 1: Fig. S1 and S2, respectively, and the findings of all quantitative meta-analyses are summarized in Table 2.

**Table 2:** Summary of the outcomes of each meta-analysis. Significant *P* values of the effect size are in bold

	Measured SCFA	Number of Studies	Heterogeneity (***I***^***2***^ %, ***P*** value)	Statistical Model	Effect size (SMD [95% CI], ***P*** value)
**CRC risk**	Acetic acid	3	99, < 0.00001	Random effect	2.63 [− 1.82 to 7.08], 0.25
	Propionic acid	3	99, < 0.00001	Random effect	2.33 [− 2.33 to 7.00], 0.33
	Butyric acid	5	99, < 0.00001	Random effect	1.47 [− 0.80 to 3.73], 0.2
	Combined^a^	11	99, < 0.00001	Random effect	2.02 [0.31 to 3.74], **0.02**
	Total SCFA	3	38, 0.2	Random effect	0.29 [− 0.13 to 0.72], 0.17
	Total SCFA	3	38, 0.2	Fixed effect	0.25 [− 0.05 to 0.56], 0.11
**CRC incidence**	Acetic acid	4	60, 0.06	Random effect	0.61 [0.09 to 1.13], **0.02**
	Propionic acid	4	62, 0.05	Random effect	0.32 [− 0.21 to 0.84], 0.24
	Butyric acid	4	43, 0.15	Random effect	0.45 [0.02 to 0.88], **0.04**
	Combined^a^	12	51, 0.02	Random effect	0.45 [0.19 to 0.72], **0.0009**
	Butyric acid	4	43, 0.15	Fixed effect	0.42 [0.1 to 0.74], **0.009**

^a^Combined effect size of acetic, propionic, and butyric acid. Note that the total SCFA indicates the collection of all the SCFA molecules—not only acetic, propionic, and butyric acid

Qualitative analysis was carried out on the studies which reported lower, higher or no changes to the concentration of SCFAs between high-risk CRC (for risk category) or CRC case (for incidence) and low-risk or control, respectively [23–25, 27, 29, 31, 32, 34–40] (Additional file 1: Fig. S3). In the risk category, more studies (70.4%) reported significantly lower concentrations of fecal acetic, propionic, and butyric acid as well as total SCFA in individuals at high risk of CRC. In the incidence category, more studies (66.7%) reported significantly lower concentrations of fecal acetic and butyric acid in CRC patients compared to healthy controls. However, the number of studies reporting no significant difference in the propionic acid was the highest in the incidence category. Overall, our qualitative analysis (Additional file 1: Fig. S3) corroborates with the meta-analysis results (Fig. 2).

## Discussion

For more than three decades, in vitro, animal, and human studies have identified numerous potentially beneficial anti-inflammatory and anti-carcinogenic roles of SCFA molecules in gut health and colonic diseases [6, 14–17, 53]. In addition, several meta-analyses (Additional file 1: Table S4) have assessed the role of colonic microbiota [54], non-digestible carbohydrates [55] and dietary fiber in colorectal carcinoma [11, 56] or adenoma [9, 10] as well as the alteration of SCFAs in irritable bowel syndrome (IBS) [57], or inflammatory bowel disease (IBD) [58].

This systematic review and meta-analysis were conducted on 23 studies to better determine the potential association between fecal SCFA concentration and CRC risk and incidence. The combined mean difference of acetic, propionic, and butyric acid in the CRC risk category analysis revealed a significantly lower concentration of these SCFAs in individuals at risk of developing CRC compared to healthy subjects, indicating a potential association between these three major SCFA molecules and CRC development. This finding was further confirmed in the CRC incidence category analysis where the fecal SCFA levels in CRC patients were significantly lower compared to those in healthy subjects.

Our findings in CRC risk and incidence were consistent with the observations reported in other meta-analyses, which focused on the association between dietary fiber intake and the risk of colorectal adenoma [9, 10], and carcinoma [11]. These systematic reviews suggested a protective effect of dietary fiber intake against CRA and CRC [9–11]. Since SCFAs are produced by gut-microbiota via the fermentation of dietary fibers [14–17], our meta-analysis of SCFA concentrations in CRC further confirms earlier observations and underlines the importance of dietary fibers/SCFAs in the risk and progression of CRC.

Another meta-analysis, which assessed the effect of non-digestible carbohydrate [resistance starch (RS)] or inulin supplementation on the risk of colorectal neoplasia, did not find significant increase in fecal total SCFA or butyric acid concentration and excretion before and after the intervention [55]. Many studies which investigated the effect of RS on healthy subjects or individuals with sporadic CRC or adenoma had a period of ≤ 4-week of intervention. A few studies reported 7- and 8-week intervention on adenoma or healthy individuals and the remaining studies were conducted on individuals with inherited CRC syndromes after > 2-year intervention [55]. The duration of intervention was longest (> 2 years) for studies involving hereditary CRC cases with reported germ-line mutations, which may have outweighed the effect of RS supplementation, while interventions involving sporadic cases or healthy subjects had much shorter periods of RS intervention (< 8 weeks) [55]. In our meta-analysis, we also did not observe a significant difference in total fecal SCFAs in the CRC risk category. This could be due to other SCFA molecules such as valeric, iso-butyric, and iso-valeric acid being included in total SCFA measurements; the latter two are the branched SCFAs mainly produced via fermentation of branched amino acids in the colon and not from non-digestible carbohydrates [59, 60].

Another systematic review on the food-microorganism-SCFA axis, without any meta-analysis, concluded that most evidence demonstrated higher SCFA levels in individuals at risk of CRC compared to healthy individuals [61], which contrasts with findings in our systematic review which showed lower fecal SCFA concentration in at-risk individuals (Fig. 2). In comparison to our systematic review, their search strategy restricted their analysis to only 8 of the final 23 studies that we analyzed [19, 27, 31, 32, 37–40]. Therefore, their conclusion was based on a smaller subset of the primary studies available and was also not supported by a meta-analysis.

Both the quantitative and qualitative analyses of CRC risk identified comparable findings of significantly lower concentration of acetic and butyric acid in the high- versus low-risk CRC group. For the CRC incidence category, the quantitative meta-analysis of butyric acid was consistent with observations identified in most of the articles from the qualitative analysis, supporting the evidence of lower concentration of three SCFAs in CRC cases compared to healthy controls. The meta-analysis of propionic acid was not significantly different between cases and controls. Similarly, most of the studies (5 of 7) reported no significant difference in fecal propionic acid concentration between CRC and healthy control in the qualitative analysis. The meta-analysis on IBS revealed a significantly higher concentration of fecal propionic acid in these patients in comparison to healthy controls [57]. Therefore, further studies comparing SCFA profiles among multiple gut diseases could shed more light on the importance of these molecules in the development of varied medical conditions.

To our knowledge, this systematic review is the first to provide a comprehensive search and data collection on observational studies linking SCFA molecules with the CRC risk and incidence. A limitation of our analysis is the heterogeneity of the studies evaluated in this systematic review, which is very difficult to control for. One such factor was the age group assessed for CRC incidence and risk. The mean age of the group in the studies was greater than 50 years and fecal SCFA concentration was not measured in younger populations to provide a comparison with low-risk, young age individuals. Although CRC is most often diagnosed in individuals > 50 years, the incidence for early-onset CRC (EOCRC) in adults aged 20–49 years has increased over the past decade in the USA, Australia, and Europe [62–65]. It would be of interest in the future to study different age group populations for CRC risk and incidence. Family history [66], diet, and lifestyle [67] are known factors contributing to CRC incidence. Only a few studies assessed in this systematic review provided information on the dietary difference between groups [19, 21, 35, 36, 40]. There was also no information about the type of polyps (conventional vs serrated) in individuals with CRA.

One common limitation is related to the nature of observational studies. Case-control studies are inherently prone to recall bias and appropriate matching of case and control groups [68]. Similarly, the results of the cross-sectional studies could be affected by bidirectional relationship [69] and confounding factors. We have assessed the effect of these inherent limitations on our analysis by undertaking appropriate quality checks such as the “comparability” category of NOS quality assessment (Additional file 1: Table S1) and the JBI tool (Additional file 1: Table S2) for all the studies included in our systematic review and meta-analysis.

Another limitation is the diversity in sample handling/storage workflows and the methodologies used to measure fecal SCFA across the studies (Table 1). We did not have enough studies to perform separate meta-analyses to understand the effect of each of these variables on the SCFA concentrations. While these factors could influence our interpretation, the levels of SCFAs were lower in high-risk as well as incident CRC cases, irrespective of the method used to measure SCFAs. Since these are well-known and established techniques for measuring SCFA concentrations, it appears that the standardized sample handling workflows and analytical methods had little impact on differences across study groups, so long as optimized procedures for SCFA assessment were followed. This systematic review did not include non-English records. To our knowledge, no longitudinal studies have reported fecal SCFA measurements at different time points during CRC progression, nonetheless, the 23 studies assessed in this systematic review and meta-analysis provide a comparison between CRC risk/incidence and respective controls from various countries and ethnic groups.

In addition to the SCFAs assessed in this systematic review, other metabolites such as bile acids were also measured in six of the selected studies [20, 30, 32, 35, 36, 39]. Among the bile acids investigated, a significantly higher fecal concentration of deoxycholic acid in the CRC high- versus low-risk group was reported in three studies [35, 36, 39]. Dietary fiber and fat promote the production of SCFA and bile acid molecules in the gut, respectively, and the latter is associated with gastrointestinal carcinogenesis [70–72]. Measurement of fecal SCFAs and other gut metabolites (such as bile acids) in longitudinal studies comparing individuals with colorectal adenoma/risk and healthy subjects could strengthen their association with CRC progression.

This study supports further exploration into fecal concentration of SCFAs: acetic, propionic, and butyric acids, as biomarkers for CRC risk. Among the current CRC screening methods, colonoscopy is the gold standard [73]; however, being invasive, it presents some procedural risk [74]. The guaiac fecal occult blood test (gFOBT) and fecal immunochemical test (FIT) are other, in practice, non-invasive stool-based methods for CRC screening, which however require improvement, in particular for detection of CRA or early-stage colonic carcinogenesis [74–76]. Fecal SCFA could be considered as a potential non-invasive biomarker to be measured in combination with or as an alternative to the commonly used non-invasive and current CRC screening methods [74, 77], to improve specificity and sensitivity of current screening, as well as for potential early detection of CRA.

## Conclusions

Gut microbiota dysbiosis and changes in their metabolites have been the focus of epidemiological studies aimed at uncovering associations with colonic inflammation and carcinogenesis. In line with the protective role of fecal SCFAs against the development of gut diseases [15, 16], and the protective effect of dietary fibers against CRC risk and/or incidence [9–11], we determined that the combined fecal concentration of the three major SCFA molecules was significantly lower not only in CRC patients compared to healthy controls, but also in high-risk CRC individuals. Gut SCFA concentrations are inversely associated with CRC-risk as well as CRC-incidence and could be biomarkers for predicting CRC-progression, as well as a drug target (in future intervention studies) aimed to retard or prevent CRC progression.

## Registration

The study is registered in PROSPERO database (registration code: CRD42021256123).

## Supplementary Information

**Additional file 1.**

## Data Availability

All the analyses are available within an institutional repository and can be provided upon request.

## Abbreviations

CRC: Colorectal cancer
CRA: Colorectal adenoma
SCFA: Short-chain fatty acid
GLOBOCAN: Global Cancer Incidence, Mortality and Prevalence
PRISMA: Preferred Reporting Items for Systematic Reviews and Meta-Analyses
NOS: Newcastle-Ottawa Scale
JBI: Joanna Briggs Institute
RevMan: Review Manager
SMD: Standardized mean difference
RS: Resistance starch
IBS: Irritable bowel syndrome
IBD: Inflammatory bowel disease
EOCRC: Early-onset colorectal cancer
gFOBT: Guaiac fecal occult blood test
FIT: Fecal immunochemical test
