# Supplementary material for: Short-chain fatty acid concentrations in the incidence and risk-stratification of colorectal cancer: a systematic review and meta-analysis
Source: BMC Med. 2022 Oct 3;20:323. doi: 10.1186/s12916-022-02529-4 (PMC9528142; doi:10.1186/s12916-022-02529-4)
Supplement: Supplementary file 1 — Additional file 1. [file 12916_2022_2529_MOESM1_ESM.docx]

Additional file 1

**Short-chain fatty acid concentrations in the incidence and risk-stratification of colorectal cancer: a systematic review and meta-analysis**

Ehsan Alvandi^1,2^, Wilson K M Wong^1,3^, Mugdha V Joglekar^1,3^, Kevin J Spring^4,2,5,*^, Anandwardhan A Hardikar^1,3,6,*^

**Affiliations:**

^1^ Diabetes and Islet Biology Group, School of Medicine, Western Sydney University, Campbelltown, NSW, Australia

^2^ Medical Oncology Group, Ingham Institute for Applied Medical Research, Liverpool, NSW, Australia

^3^ Translational Health Research Institute, Western Sydney University, Campbelltown, NSW, Australia

^4^ Liverpool Clinical School, School of Medicine, Western Sydney University, Liverpool, NSW, Australia

^5^ South-West Sydney Clinical Campuses, UNSW Medicine & Health, Sydney, NSW, Australia

^6^ Department of Science and Environment, Roskilde University Copenhagen, Roskilde, Denmark

*Address all correspondence to:

Kevin J. Spring, PhD (k.spring@westernsydney.edu.au), or

Anandwardhan A. Hardikar, PhD (a.hardikar@westernsydney.edu.au)

**Methods: 1**

**Tables: 4**

**Figures: 3**

**Supplementary methods.** The details of the search strategy conducted on May 21, 2021.

1) Medline and Embase combined search, followed by deduplication, using Ovid search interface:

Search link:

<https://ezproxy.uws.edu.au/login?url=http://ovidsp.ovid.com/ovidweb.cgi?T=JS&NEWS=N&PAGE=main&SHAREDSEARCHID=6oHhfLqbyqUT2KwL2FpUgcWyfohUdPU40kumYqdCTlI8X2Ibo6ZjVT3zsbLZCbIw7>

Search history details:

Embase <1974 to 2021 May 20>

Ovid MEDLINE(R) ALL

1 exp Fatty Acids, Volatile/ 113151

2 short chain fatty acid*.mp. 24264

3 short-chain fatty acid*.mp. 24264

4 SCFA*.mp. 10870

5 exp Acetates/ 166777

6 acetate.mp. 427012

7 exp Propionates/ 28315

8 propionate.mp. 56255

9 exp Butyrates/ 90653

10 butyrate.mp. 35882

11 exp Colorectal Neoplasms/ 240756

12 exp Colonic Neoplasms/ 418868

13 "colorectal cancer".mp. 324884

14 "colon cancer".mp. 157155

15 "colorectal carcinoma".mp. 48725

16 "colon carcinoma".mp. 39183

17 "colorectal neoplasm".mp. 2133

18 "colon neoplasm".mp. 294

19 "colorectal neoplasia".mp. 6269

20 "colon neoplasia".mp. 499

21 "colo* cance*".mp. 450574

22 "colo* carcinom*".mp. 93299

23 "colo* neoplas*".mp. 185903

24 crc.mp. 94637

25 1 or 2 or 3 or 4 or 5 or 6 or 7 or 8 or 9 or 10 775397

26 11 or 12 or 13 or 14 or 15 or 16 or 17 or 18 or 19 or 20 or 21 or 22 or 23 or 24 650155

27 25 and 26 7563

28 ((Fatty Acids, Volatile or short chain fatty acid* or short-chain fatty acid* or SCFA* or Acetates or acetate or Propionates or propionate or Butyrates or butyrate) and (Colorectal Neoplasms or Colonic Neoplasms or "colorectal cancer" or "colon cancer" or "colorectal carcinoma" or "colon carcinoma" or "colorectal neoplasm" or "colon neoplasm" or "colorectal neoplasia" or "colon neoplasia" or "colo* cance*" or "colo* carcinom*" or "colo* neoplas*" or crc)).ti,ab. 4013

29 28 not (letter or news or comment or editorial or congresses or abstracts).pt. 4006

30 limit 29 to humans 3044

31 remove duplicates from 30 1876

32 limit 31 to english language 1811

Next, the search results were filtered by “Publication Type: Article”, resulted in 1217 records.

The search was repeated on June 29, 2022, only for articles published in 2021 and 2022, resulting in 76 new records (1217+76=1293 records in total).

2) Web of science:

ab=( ("scfa*" OR "short chain fatty acid*" OR "short-chain fatty acid*" OR acetate OR propionate OR butyrate) AND ("colo* cance*" OR "colo* carcinom*" OR "colo* neoplas*" OR crc) AND (human* OR patient* OR subject* OR individual* OR participant* OR case* OR control* OR character* person* OR people) NOT (rats OR mouse OR mice OR murine) )

Next, the search results were refined by “Document Types: Articles”, and “Languages: English”, resulted in 783 records.

The search was repeated on June 29, 2022, only for articles published in 2021 and 2022, resulting in 57 new records (783+57=840 records in total).

**Table S1.** Quality assessment of the selected case-control studies (n = 17) using Newcastle-Ottawa Scale (NOS). According to NOS guideline, one star can be given to each sector in Selection or Exposure category, and two stars for Comparability category, one per one matched factor. ^¶^Refer to the text for the definition of CRC incidence or risk category. ^§^Based on the definition of control in NOS guideline, the star was given only to studies that “explicitly stated that controls have no history of this outcome”. ^†^As explained in the text, the SCFA values in this study could not be converted to mean and SD, and thus were included in the qualitative analysis.

|  |  |  |  | Selection |  |  | Comparability |  | Exposure |  |  |
| --- | --- | --- | --- | --- | --- | --- | --- | --- | --- | --- | --- |
|  | **Study** | **Analysis Category**^¶^ | **Adequate definition of cases** | **Representativeness of cases** | **Selection of controls** | **Definition of controls^§^** | **Control for important factor or additional factor** | **Ascertainment of exposure** | **Same method of ascertainment for cases and controls** | **Non-response rate** | **Total scores** |
| **Qualitative data**  **(values of SCFA levels not reported)** | Sze et al. 2019 | Incidence and risk | ★ | ★ |  |  |  | ★ | ★ | ★ | 5 |
|  | Lin et al. 2019 | Incidence | ★ |  | ★ |  | ★★ | ★ | ★ | ★ | 7 |
|  | Lin et al. 2016 | Incidence | ★ | ★ | ★ |  |  | ★ | ★ | ★ | 6 |
|  | Weir et al. 2013 | Incidence | ★ |  |  |  |  | ★ | ★ | ★ | 4 |
|  | Ohigashi et al. 2013 | Incidence and risk | ★ | ★ | ★ |  |  | ★ | ★ | ★ | 6 |
|  | Monleon et al. 2009 | Incidence | ★ | ★ | ★ |  |  | ★ | ★ | ★ | 6 |
|  | Weaver et al. 1988 | Incidence | ★ | ★ | ★ | ★ |  | ★ | ★ | ★ | 7 |
| **Quantitative data**  **(reported SCFA values)** | Nannini et al. 2021^†^ | Incidence and risk | ★ | ★ | ★ |  |  | ★ | ★ | ★ | 6 |
|  | Chen et al. 2021 | Risk |  | ★ | ★ |  |  | ★ | ★ | ★ | 5 |
|  | Torii et al. 2019 | Incidence | ★ |  |  |  |  | ★ | ★ | ★ | 4 |
|  | Niccolai et al. 2019 | Incidence and risk | ★ | ★ | ★ |  |  | ★ | ★ | ★ | 6 |
|  | Yusuf et al. 2018 | Incidence | ★ | ★ | ★ |  |  | ★ | ★ | ★ | 6 |
|  | Song et al. 2018 | Incidence | ★ | ★ | ★ | ★ | ★ | ★ | ★ | ★ | 8 |
|  | Bridges et al. 2018 | Risk | ★ | ★ | ★ | ★ |  | ★ | ★ | ★ | 7 |
|  | Chen et al. 2013 | Risk | ★ | ★ | ★ |  |  | ★ | ★ | ★ | 6 |
|  | Boutron-Ruault et al. 2005 | Risk | ★ | ★ | ★ | ★ | ★★ | ★ | ★ | ★ | 9 |
|  | Kashtan et al. 1992 | Risk | ★ | ★ | ★ |  |  | ★ | ★ | ★ | 6 |

**Table S2.** Quality assessment of the included cross-sectional studies (n = 6) using Joanna Briggs Institute (JBI) Critical Appraisal tool. The studies are shown with alternate shading. ^¶^Matched for sex. ^§^Matched for age and sex. ^†^Matched for age, as recruited subjects aged 50-60 years old.

| **Ocvirk et al. 2020** | Response options | | | |
| --- | --- | --- | --- | --- |
| 1. Were the criteria for inclusion in the sample clearly defined? | ***Yes*** | No | Unclear | Not applicable |
| 2. Were the study subjects and the setting described in detail? | ***Yes*** | No | Unclear | Not applicable |
| 3. Was the exposure measured in a valid and reliable way? | ***Yes*** | No | Unclear | Not applicable |
| 4. Were objective, standard criteria used for measurement of the condition? | ***Yes*** | No | Unclear | Not applicable |
| 5. Were confounding factors identified? | ***Yes*** | No | Unclear | Not applicable |
| 6. Were strategies to deal with confounding factors stated? | Yes | ***No*** | Unclear | Not applicable |
| 7. Were the outcomes measured in a valid and reliable way? | ***Yes*** | No | Unclear | Not applicable |
| 8. Was appropriate statistical analysis used? | ***Yes*** | No | Unclear | Not applicable |
| Overall appraisal: Include ☑ Exclude □ Seek further info □ | | | | |
| **Katsidzira et al. 2019** | Response options | | | |
| 1. Were the criteria for inclusion in the sample clearly defined? | ***Yes*** | No | Unclear | Not applicable |
| 2. Were the study subjects and the setting described in detail? | ***Yes*** | No | Unclear | Not applicable |
| 3. Was the exposure measured in a valid and reliable way? | ***Yes*** | No | Unclear | Not applicable |
| 4. Were objective, standard criteria used for measurement of the condition? | ***Yes*** | No | Unclear | Not applicable |
| 5. Were confounding factors identified? | ***Yes*** | No | Unclear | Not applicable |
| 6. Were strategies to deal with confounding factors stated? | ***Yes***^¶^ | No | Unclear | Not applicable |
| 7. Were the outcomes measured in a valid and reliable way? | ***Yes*** | No | Unclear | Not applicable |
| 8. Was appropriate statistical analysis used? | ***Yes*** | No | Unclear | Not applicable |
| Overall appraisal: Include ☑ Exclude □ Seek further info □ | | | | |
| **Hester et al. 2015** | Response options | | | |
| 1. Were the criteria for inclusion in the sample clearly defined? | ***Yes*** | No | Unclear | Not applicable |
| 2. Were the study subjects and the setting described in detail? | Yes | ***No*** | Unclear | Not applicable |
| 3. Was the exposure measured in a valid and reliable way? | ***Yes*** | No | Unclear | Not applicable |
| 4. Were objective, standard criteria used for measurement of the condition? | Yes | ***No*** | Unclear | Not applicable |
| 5. Were confounding factors identified? | ***Yes*** | No | Unclear | Not applicable |
| 6. Were strategies to deal with confounding factors stated? | Yes | ***No*** | Unclear | Not applicable |
| 7. Were the outcomes measured in a valid and reliable way? | Yes | No | ***Unclear*** | Not applicable |
| 8. Was appropriate statistical analysis used? | ***Yes*** | No | Unclear | Not applicable |
| Overall appraisal: Include ☑ Exclude □ Seek further info □ | | | | |
| **Ou et al. 2013** | Response options | | | |
| 1. Were the criteria for inclusion in the sample clearly defined? | ***Yes*** | No | Unclear | Not applicable |
| 2. Were the study subjects and the setting described in detail? | ***Yes*** | No | Unclear | Not applicable |
| 3. Was the exposure measured in a valid and reliable way? | ***Yes*** | No | Unclear | Not applicable |
| 4. Were objective, standard criteria used for measurement of the condition? | ***Yes*** | No | Unclear | Not applicable |
| 5. Were confounding factors identified? | ***Yes*** | No | Unclear | Not applicable |
| 6. Were strategies to deal with confounding factors stated? | ***Yes***^§^ | No | Unclear | Not applicable |
| 7. Were the outcomes measured in a valid and reliable way? | ***Yes*** | No | Unclear | Not applicable |
| 8. Was appropriate statistical analysis used? | ***Yes*** | No | Unclear | Not applicable |
| Overall appraisal: Include ☑ Exclude □ Seek further info □ | | | | |
| **Ou et al. 2012** | Response options | | | |
| 1. Were the criteria for inclusion in the sample clearly defined? | ***Yes*** | No | Unclear | Not applicable |
| 2. Were the study subjects and the setting described in detail?  (According to this reference of the paper: O’Keefe et al. 2007, PMID: 17182822.) | ***Yes*** | No | Unclear | Not applicable |
| 3. Was the exposure measured in a valid and reliable way? | ***Yes*** | No | Unclear | Not applicable |
| 4. Were objective, standard criteria used for measurement of the condition? | ***Yes*** | No | Unclear | Not applicable |
| 5. Were confounding factors identified? | ***Yes***^†^ | No | Unclear | Not applicable |
| 6. Were strategies to deal with confounding factors stated? | Yes | ***No*** | Unclear | Not applicable |
| 7. Were the outcomes measured in a valid and reliable way? | ***Yes*** | No | Unclear | Not applicable |
| 8. Was appropriate statistical analysis used? | ***Yes*** | No | Unclear | Not applicable |
| Overall appraisal: Include ☑ Exclude □ Seek further info □ | | | | |
| **O’Keefe et al. 2009** | Response options | | | |
| 1. Were the criteria for inclusion in the sample clearly defined? | ***Yes*** | No | Unclear | Not applicable |
| 2. Were the study subjects and the setting described in detail?  (Only the time period of living in either area wasn’t mentioned.) | ***Yes*** | No | Unclear | Not applicable |
| 3. Was the exposure measured in a valid and reliable way?  (According to this reference of the paper: O’Keefe et al. 2007, PMID: 17182822.) | ***Yes*** | No | Unclear | Not applicable |
| 4. Were objective, standard criteria used for measurement of the condition? | ***Yes*** | No | Unclear | Not applicable |
| 5. Were confounding factors identified? | ***Yes***^†^ | No | Unclear | Not applicable |
| 6. Were strategies to deal with confounding factors stated? | Yes | ***No*** | Unclear | Not applicable |
| 7. Were the outcomes measured in a valid and reliable way? | ***Yes*** | No | Unclear | Not applicable |
| 8. Was appropriate statistical analysis used? | ***Yes*** | No | Unclear | Not applicable |
| Overall appraisal: Include ☑ Exclude □ Seek further info □ | | | | |

**Table S3.** Table showing which data is used for which analysis. Cross-sectional studies are highlighted in grey, and case-control studies are not highlighted. Total number of studies in each category is stated at the bottom of table. Note: for quantitative analyses in risk category, the meta-analyses in the **Figure 2** included 3, 3, 5, and 3 studies for measuring C2, C3, C4, and total SCFA, respectively. C2: acetic acid, C3: propionic acid, C4: butyric acid. ^¶^As explained in the text, the SCFA values in this study could not be converted to mean and SD, and thus were included in the qualitative analysis.

|  |  | **CRC risk** | | | | **CRC incidence** | | | |
| --- | --- | --- | --- | --- | --- | --- | --- | --- | --- |
|  |  | **C2** | **C3** | **C4** | **Total SCFA** | **C2** | **C3** | **C4** | **Total SCFA** |
| **Qualitative data (values of SCFA levels not reported)** | Sze et al. 2019 | * | * | * |  | * | * | * |  |
|  | Lin et al. 2019 |  |  |  |  | * | * | * |  |
|  | Lin et al. 2016 |  |  |  |  | * | * | * |  |
|  | Weir et al. 2013 |  |  |  |  | * | * | * |  |
|  | Ohigashi et al. 2013 | * |  |  |  | * |  |  |  |
|  | Monleon et al. 2009 |  |  |  |  | * | * | * |  |
|  | Weaver et al. 1988 |  |  |  |  | * | * | * | * |
|  | Ocvirk et al. 2020 | * | * |  | * |  |  |  |  |
|  | Katsidzira et al. 2019 | * | * | * |  |  |  |  |  |
|  | Hester et al. 2015 | * | * | * | * |  |  |  |  |
|  | Ou et al. 2013 | * | * | * |  |  |  |  |  |
|  | Ou et al. 2012 | * | * | * |  |  |  |  |  |
|  | O’Keefe et al. 2009 | * | * | * | * |  |  |  |  |
| **Quantitative data (reported SCFA values)** | Nannini et al. 2021^¶^ | * | * | * |  | * | * | * |  |
|  | Chen et al. 2021 | * | * | * |  |  |  |  |  |
|  | Torii et al. 2019 |  |  |  |  | * | * | * |  |
|  | Niccolai et al. 2019 | * | * | * | * | * | * | * | * |
|  | Yusuf et al. 2018 |  |  |  |  | * | * | * |  |
|  | Song et al. 2018 |  |  |  |  | * | * | * |  |
|  | Bridges et al. 2018 | * | * | * | * |  |  |  |  |
|  | Chen et al. 2013 | * | * | * |  |  |  |  |  |
|  | Boutron-Ruault et al. 2005 | * | * | * | * |  |  |  |  |
|  | Kashtan et al. 1992 |  |  | * | * |  |  |  |  |
|  | Ocvirk et al. 2020 |  |  | * |  |  |  |  |  |
|  | Total number of studies,  in **qualitative** data analysis | **9** | **8** | **7** | **3** | **8** | **7** | **7** | **1** |
|  | Total number of studies,  in **quantitative** data analysis | **5** | **5** | **7** | **4** | **4** | **4** | **4** | **1** |
|  |  | **C2** | **C3** | **C4** | **Total SCFA** | **C2** | **C3** | **C4** | **Total SCFA** |
|  |  | **CRC risk** | | | | **CRC incidence** | | | |

**Table S4.** The summary of other systematic reviews related to fibre intake, SCFA and risk of colorectal cancer or adenoma.

| **Study** | **PMID** | **Title** | **Aim** | **Primary source** | **Meta-analysis** | **Outcome** |
| --- | --- | --- | --- | --- | --- | --- |
| Rao et al. 2021 | 32202158 | Non-Digestible Carbohydrate and the Risk of Colorectal Neoplasia: A Systematic Review | To check whether RS and inulin should be offered to cancer/precancerous patients or healthy subjects to decrease their risk of CRC. | Interventional studies on resistant starch (RS) or inulin supplementation | Total SCFA and butyrate concentration and excretion | Total SCFAs and butyrate concentrations and excretions in feces did not increase significantly after RS/inulin supplementation. |
| Nucci et al. 2021 | 33920845 | Association between Dietary Fibre Intake and Colorectal Adenoma: A Systematic Review and Meta-Analysis | Assessing the association between dietary fibre intake and the risk of colorectal adenoma in adults. | Interview and questionnaire-based case-control, cohort, and cross-sectional studies | Effect size is measured as odds ratio | There could be a protective effect of dietary fibre intake against colorectal adenoma. |
| Shuwen et al. 2019 | 31401674 | Protective effect of the "food-microorganism-SCFAs" axis on colorectal cancer: from basic research to practical application | To elucidate the “food-microorganism-SCFAs” axis and to provide guidance for prevention and intervention in CRC. | Not specific. Human, animal, and cell-based studies | - | The concentrations of SCFAs in CRC patients and individuals with a high risk of CRC were higher than those in healthy individuals. |
| Oh et al. 2019 | 31495339 | Different dietary fibre sources and risks of colorectal cancer and adenoma: a dose-response meta-analysis of prospective studies | To summarise the relationships of different fibre sources with colorectal cancer and adenoma risks. | Interview and questionnaire-based prospective cohort studies | Effect size is measured as relative risk | The evidence for colorectal cancer prevention is strongest for fibre from cereals/grains. Each 10 g/d increase in dietary intake of vegetable or fruit fibre was statistically significantly associated with a reduced risk of incident colorectal adenoma. |
| Gianfredi et al. 2018 | 29516760 | Is dietary fibre truly protective against colon cancer? A systematic review and meta-analysis | To evaluate the association between dietary fibre intake and the risk of colon cancer. | Interview and questionnaire-based case-control, cohort, and cross-sectional studies | Effect size is measured as odds ratio | Results suggest a protective role of dietary fibre intake on colon cancer risk. |


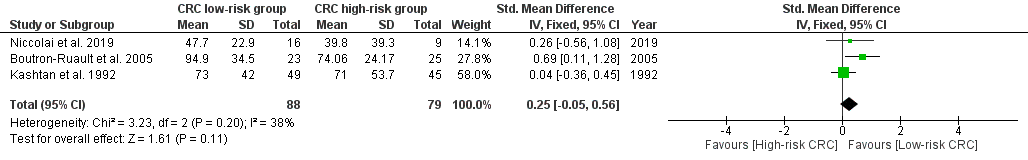


**Fig. S1.** Forest plot representing the meta-analyses of the fecal total SCFA concentration in the CRC risk category using fixed-effect model. Note that total SCFA indicates the collection of all the SCFA molecules - not only acetic, propionic, and butyric acid.


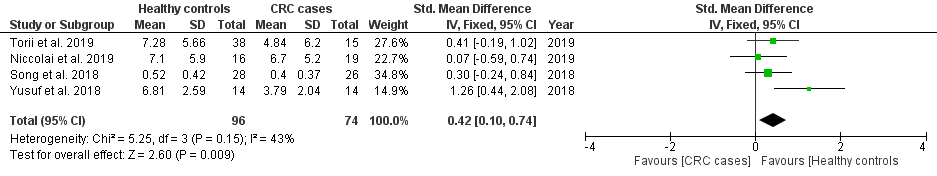


**Fig. S2.** Forest plot representing the meta-analyses of the fecal butyric acid concentration in the CRC incidence category using fixed-effect model.


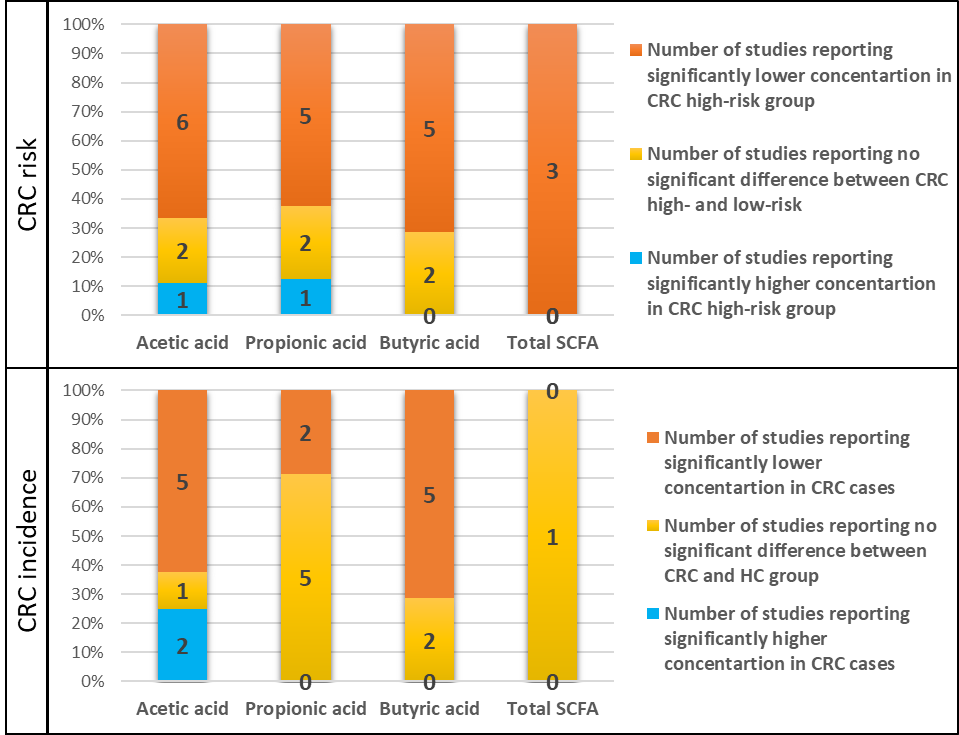


**Fig. S3.** Graphical representation of fecal SCFA concentration. Stacked bar charts summarizing the results of the qualitative data in CRC risk (9, 8, 7, and 3 studies have measured fecal concentration of acetic, propionic, and butyric acid, and total SCFA, respectively) and incidence categories (8, 7, 7, and 1 study have measured fecal concentration of acetic, propionic, and butyric acid, and total SCFA, respectively). HC: healthy controls.
